# A highly efficient sulfadiazine selection system for the generation of transgenic plants and algae

**DOI:** 10.1111/pbi.13004

**Published:** 2018-09-13

**Authors:** Iman Tabatabaei, Cristina Dal Bosco, Marta Bednarska, Stephanie Ruf, Jörg Meurer, Ralph Bock

**Affiliations:** ^1^ Max‐Planck‐Institut für Molekulare Pflanzenphysiologie Potsdam‐Golm Germany; ^2^ Department für Biologie I Ludwig‐Maximilians‐Universität München München Germany; ^3^ Present address: Pioneer Hi‐Bred Northern Europe Service Division GmbH Eschbach Germany

**Keywords:** selectable marker, plant transformation, folate biosynthesis, sulfadiazine selection, mitochondrion, transgenic plant, *Nicotiana tabacum*, *Chlamydomonas reinhardtii*

## Abstract

The genetic transformation of plant cells is critically dependent on the availability of efficient selectable marker gene. Sulfonamides are herbicides that, by inhibiting the folic acid biosynthetic pathway, suppress the growth of untransformed cells. Sulfonamide resistance genes that were previously developed as selectable markers for plant transformation were based on the assumption that, in plants, the folic acid biosynthetic pathway resides in the chloroplast compartment. Consequently, the Sul resistance protein, a herbicide‐insensitive dihydropteroate synthase, was targeted to the chloroplast. Although these vectors produce transgenic plants, the transformation efficiencies are low compared to other markers. Here, we show that this inefficiency is due to the erroneous assumption that the folic acid pathway is located in chloroplasts. When the RbcS transit peptide was replaced by a transit peptide for protein import into mitochondria, the compartment where folic acid biosynthesis takes place in yeast, much higher resistance to sulfonamide and much higher transformation efficiencies are obtained, suggesting that current *sul* vectors are likely to function due to low‐level mistargeting of the resistance protein to mitochondria. We constructed a series of optimized transformation vectors and demonstrate that they produce transgenic events at very high frequency in both the seed plant tobacco and the green alga *Chlamydomonas reinhardtii*. Co‐transformation experiments in tobacco revealed that *sul* is even superior to *nptII*, the currently most efficient selectable marker gene, and thus provides an attractive marker for the high‐throughput genetic transformation of plants and algae.

## Introduction

Marker genes are essential tools for genetic transformation. They allow the efficient identification of transgenic events by distinguishing transformed cells from non‐transformed cells, usually by killing untransformed cells or inhibiting their growth (positive selectable markers). Typical marker genes confer resistances to antibiotics or herbicides, or complement metabolic deficiencies of specific (auxotrophic) mutants. Most resistance genes encode either enzymes that detoxify the selection agent or variants of the target protein of the selection agent that confer insensitivity, typically by lacking the binding site for the inhibiting substance. A prominent example of the latter mode of resistance is provided by sulfonamides, a class of drugs and herbicides that inhibit a key protein in folate (vitamin B9) metabolism, the enzyme dihydropteroate synthase (DHPS; Brown, 1962).

Tetrahydrofolate (THF) is an essential co‐factor of metabolic enzymes in all organisms. As a donor of one‐carbon (C_1_) units, it is involved in biochemical reactions that form, for example, amino acids (methionine, glycine and serine), nucleotides, several vitamins and the initiator tRNA charged with N‐formylmethionine (Hanson and Gregory, 2011). THF is a tripartite molecule, composed of pteridine, *p*‐amino benzoate (*p‐*ABA) and glutamate moieties. The enzyme dihydropteroate synthase (DHPS) joins 6‐hydroxymethyldihydropterin pyrophosphate (derived from GTP) with *p*‐aminobenzoic acid (*p*‐ABA; synthesized from chorismate via the shikimate pathway) to form dihydropteroate (Hanson and Gregory, 2002, 2011). Sulfonamides are structural analogs of *p‐*ABA and act as competitive inhibitors of DHPS. Bacteria, plants and other organisms that depend on *de novo* synthesis of folate (de Crécy‐Lagard *et al*., 2007) are sensitive to sulfonamides. By contrast, animals acquire folate through the diet, lack the folate biosynthetic pathway and, therefore, are insensitive to sulfonamides.

Due to the essentiality of the folate pathway in bacteria and plants, sulfonamides are extensively used as antibiotics to control bacterial infections and as herbicides for weed control. In addition, sulfonamides can be used as selection agents in plant transformation (Guerineau *et al*., 1990a; Wallis *et al*., 1996; Hadi *et al*., 2002; Thomson *et al*., 2011). Resistance to sulfonamides is conferred by DHPS variants that are insensitive to the drug. For example, the *sul* gene isolated from R plasmids of Enterobacteriaceae encodes a DHPS that is insensitive to inhibition by sulfonamides (Guerineau *et al*., 1990a,b). When the *sul* gene was tested as a potential selectable marker gene for plant transformation, it was assumed that the folate pathway in plants resides in the chloroplast. This assumption was based on circumstantial genetic evidence (Smith *et al*., 1991; Wallis *et al*., 1996) and the knowledge that shikimate and *p*‐ABA synthesis occur in the plastid compartment (Hanson and Gregory, 2002, 2011). Therefore, in all transformation vectors, the *sul* coding sequence was fused to the transit peptide from the Rubisco small subunit of pea (*Pisum sativum*) to target the Sul protein (i.e., the sulfonamide‐resistant DHPS) to the chloroplast. The successful generation of transgenic plants with these constructs in a number of plant species (Guerineau *et al*., 1990a; Wallis *et al*., 1996; Hadi *et al*., 2002; Thomson *et al*., 2011) seemed to confirm the chloroplast localization of DHPS and the entire folate pathway. However, subsequently obtained evidence for a mitochondrial localization of DHPS and the downstream enzymes of the pathway has cast considerable doubt on the chloroplast location of the folate pathway in plants (Rébeillé *et al*., 1997; Hanson and Gregory, 2002, 2011). It is now generally believed that *p*‐ABA is exported from the plastid and imported into mitochondria where it is joined with 6‐hydroxymethyldihydropterin pyrophosphate by DHPS and where also all subsequent steps in folate biosynthesis take place.

Here we have attempted to resolve the discrepancy between the suspected mitochondrial localization of folate biosynthesis and the sulfonamide resistance conferred by plastid‐targeted drug‐resistant DHPS enzymes. We show that much higher levels of sulfonamide resistance are obtained when DHPS is targeted to the mitochondrial compartment. Our data suggest that current *sul*‐based transformation vectors are likely to work only due to low‐level mistargeting of the resistance protein to mitochondria. Importantly, a new set of vectors containing optimized *sul* cassettes gives rise to exceptionally high transformation frequencies that, in tobacco, are rivalled only by the kanamycin resistance gene *nptII*, the currently most efficient selectable marker gene for plant transformation. Finally, we demonstrate that optimized *sul* vectors also provide an efficient selectable marker for the transformation of the model green alga *Chlamydomonas*. Thus, *sul*‐based vectors that target the resistance protein to mitochondria offer great potential as a nearly universal selectable marker gene for the development of efficient transformation protocols for plants and algae.

## Results

### Optimization of the sulfadiazine selection system for tobacco

To determine the minimum concentration of sulfadiazine that allows selection of *sul*‐expressing cells (by suppression of the growth of wild‐type cells), a series of sensitivity tests and biolistic transformation experiments were conducted in tobacco. Low concentrations of sulfadiazine (25 mg/L) were sufficient to inhibit the growth of wild‐type cells (Figure S1). For initial transformation experiments, vector p35S*sul* was used. It contains the *sul* cassette (driven by the CaMV 35S promoter and the RbcS transit peptide) that was used in all previous transformation studies that employed sulfadiazine selection (Guerineau *et al*., 1990a; Wallis *et al*., 1996; Hadi *et al*., 2002; Thomson *et al*., 2011; Dal Bosco *et al*., 2004). These experiments confirmed that transgenic plants can be obtained by selection for 20 or 25 mg/L sulfadiazine. However, under these conditions, a substantial background of regenerating non‐transgenic clones was seen. This background could be suppressed by performing a medium change after 2–3 weeks indicating that depletion and/or decomposition of the selection agent was the cause of the escapes.

**Figure 1:**
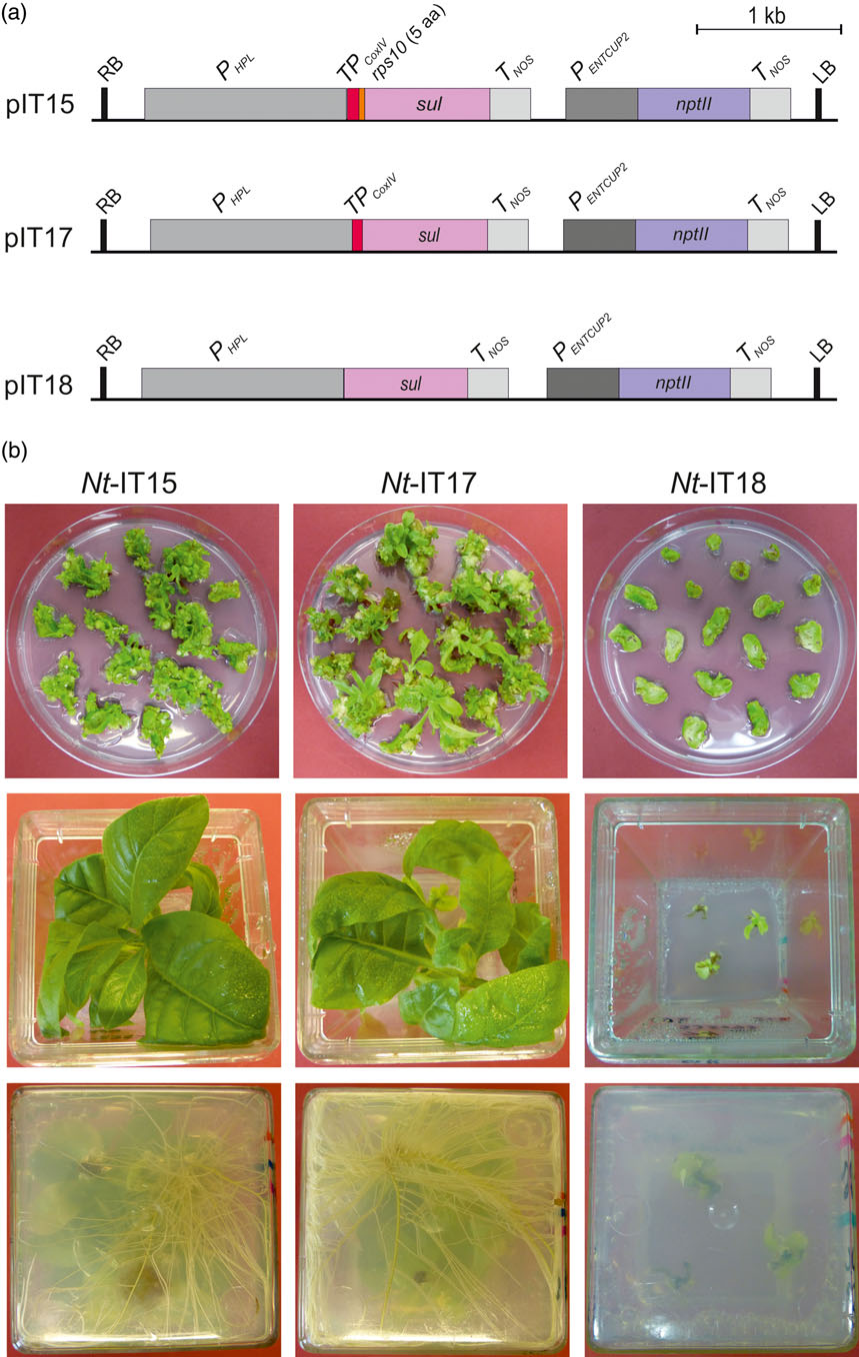
Construction of nuclear transformation vectors for optimization of the sulfadiazine selection system and generation of transgenic tobacco lines. (a) Map of the T‐DNA transfer regions in nuclear transformation vectors pIT15, pIT17 and pIT18. pIT15 and pIT17 target the Sul protein to mitochondria by the CoxIV transit peptide (red). The *sul* gene in pIT15 additionally encodes the 5 N‐terminal codons of *rps10* (orange). Vector pIT18 contains a *sul* gene without a transit peptide. Pink and blue boxes represent the *sul* and *nptII* selectable marker genes, respectively. Expression elements (promoters, terminators) are shown as grey boxes, the left (LB) and right borders (RB) of the T‐DNA are also indicated. (b) Regeneration of primary sulfadiazine‐resistant lines (upper row; photos taken 3 weeks after Agrobacterium‐mediated transformation). Sulfadiazine‐resistant shoots from pIT15 and pIT17 selection plates continue to grow after transfer to hormone‐free sulfadiazine‐containing medium (middle row) and quickly develop roots (bottom row). By contrast, shoots from pIT18 plates grow very poorly on sulfadiazine‐containing medium and are unable to root (photos taken after 5 weeks).

Based on the published evidence for mitochondrial localization (Rébeillé *et al*., 1997), we assumed that the initially proposed chloroplast localization of the folate pathway in plants was incorrect and the generation of transgenic plants by sulfadiazine selection can be substantially improved by targeting the resistance protein to mitochondria. To assess whether mitochondrial targeting facilitates selection of transgenic plants, two *sul* cassettes with mitochondrial targeting signals were constructed (Figure 1a). In both vectors (pIT15 and pIT17), mitochondrial targeting is achieved by the transit peptide sequence from the yeast *CoxIV* gene that was shown previously to confer efficient targeting to plant mitochondria (Köhler *et al*., 1997). In vector pIT15, the Sul protein additionally harbours the first five amino acids of the mitochondrial Rps10 protein. As the N‐terminus is known to be an important determinant of protein stability in organelles (Vögtle *et al*., 2009; Apel *et al*., 2010; Elghabi *et al*., 2011), the N‐terminus of a native mitochondrial protein was used to minimize the risk of Sul protein instability inside plant mitochondria. As a control, vector pIT18 was constructed, in which the Sul protein lacks any targeting information and, therefore, should result in cytosolic localization of the sulfonamide‐resistant DHPS.

Agrobacterium‐mediated transformation experiments with vectors pIT15 and pIT17 yielded more than 100 putative transgenic lines after 3–4 weeks of selection on sulfadiazine‐containing medium. The majority of the lines (82%; Table S1) continued to grow after transfer to fresh selection medium and quickly rooted on hormone‐free medium with sulfadiazine (Figure 1b). By contrast, none of the primary regenerating shoots obtained from transformation with vector pIT18 showed resistance after transfer to fresh medium, and none of the lines developed roots in sulfadiazine‐containing medium (Figure 1b, Table S1). These observations provided strong evidence that no true transgenic lines had been obtained with pIT18, thus indicating that cytosolically localized Sul protein does not confer sulfadiazine resistance.

The transgenic status of the lines produced with the mitochondrially targeted Sul version and the stable inheritance of the *sul* transgene were ultimately verified by reciprocal crosses and inheritance assays that revealed Mendelian segregation of the sulfadiazine resistance in the next generation (Figure S2). No significant difference in the number of transgenic events was seen between pIT15 and pIT17 (Figure 1; Table S1), indicating that the different N‐termini of the Sul protein either have no impact on protein stability or protein stability does not limit the level of resistance to sulfadiazine.

**Figure 2:**
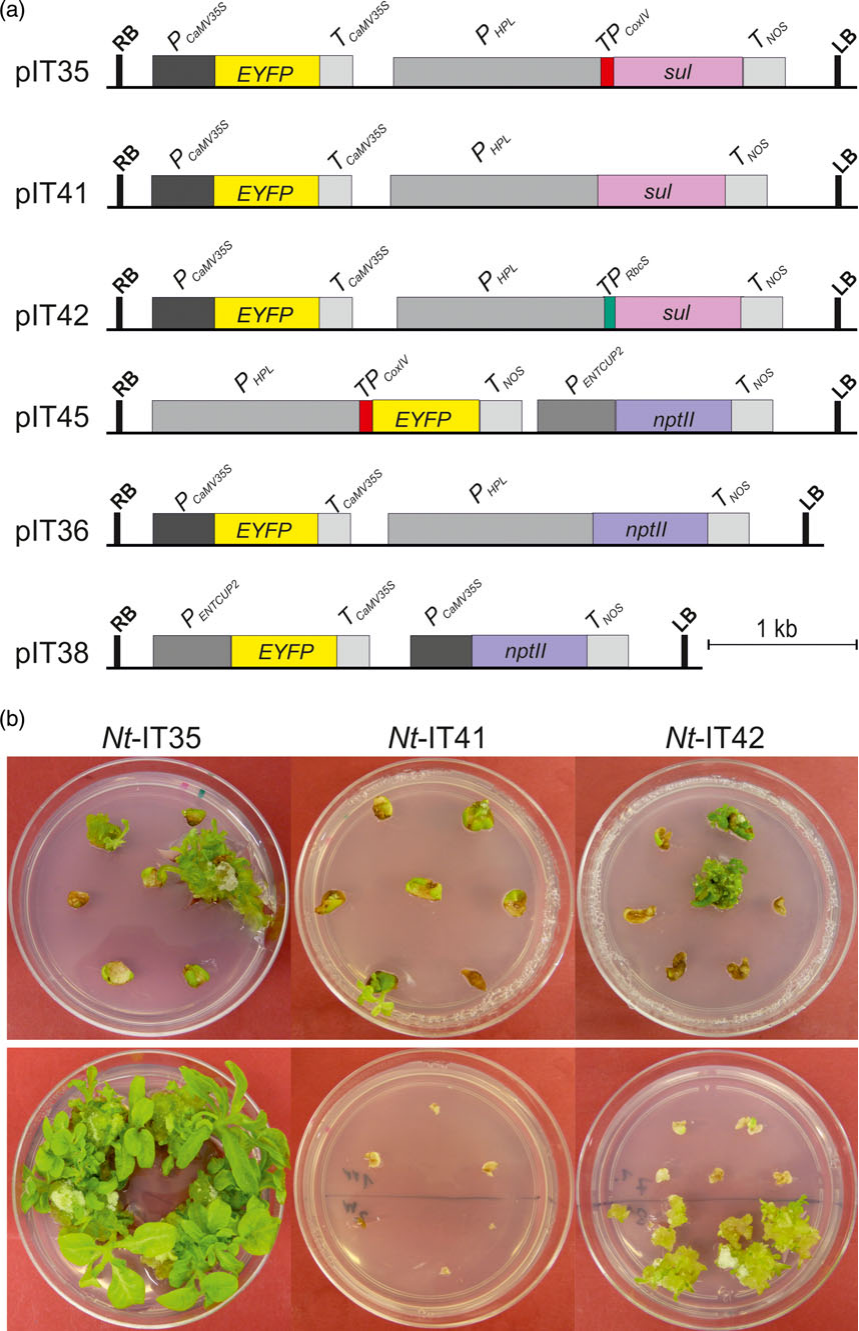
Construction of nuclear transformation vectors to confirm the specificity of the sulfadiazine selection system for mitochondria and compare the efficiency of the optimized sulfadiazine selection system with that of the kanamycin selection system. (a) Transformation vectors. Vector pIT35 contains a *sul* gene that targets the encoded resistance protein to mitochondria *via* the *CoxIV* transit peptide. Vector pIT41 contains a *sul* gene without a transit peptide. Vector pIT42 contains a *sul* variant that targets Sul to the chloroplast *via* the *RbcS* transit peptide. Vector pIT45 contains a *YFP* gene that targets the encoded protein to mitochondria *via* the CoxIV transit peptide. pIT36 and pIT38 are nuclear vectors containing the *nptII* gene driven by the *HPL* promoter (a relatively weak promoter) and the CaMV35S promoter (a very strong promoter), respectively. Pink, blue and yellow boxes represent the *sul, nptII* and *YFP* transgenes. Expression elements are shown as dark and light grey boxes. The left (LB) and right borders (RB) of the T‐DNA are also indicated. (b) Selected primary sulfadiazine‐resistant shoots and calli (top row) were transferred to an additional regeneration round (bottom panel) conducted on medium with 25 mg/L sulfadiazine. While most *Nt*‐IT35 lines and a few *Nt*‐IT42 lines continued to grow, none of the *Nt*‐IT41 lines survived the secondary selection.

**Figure 3:**
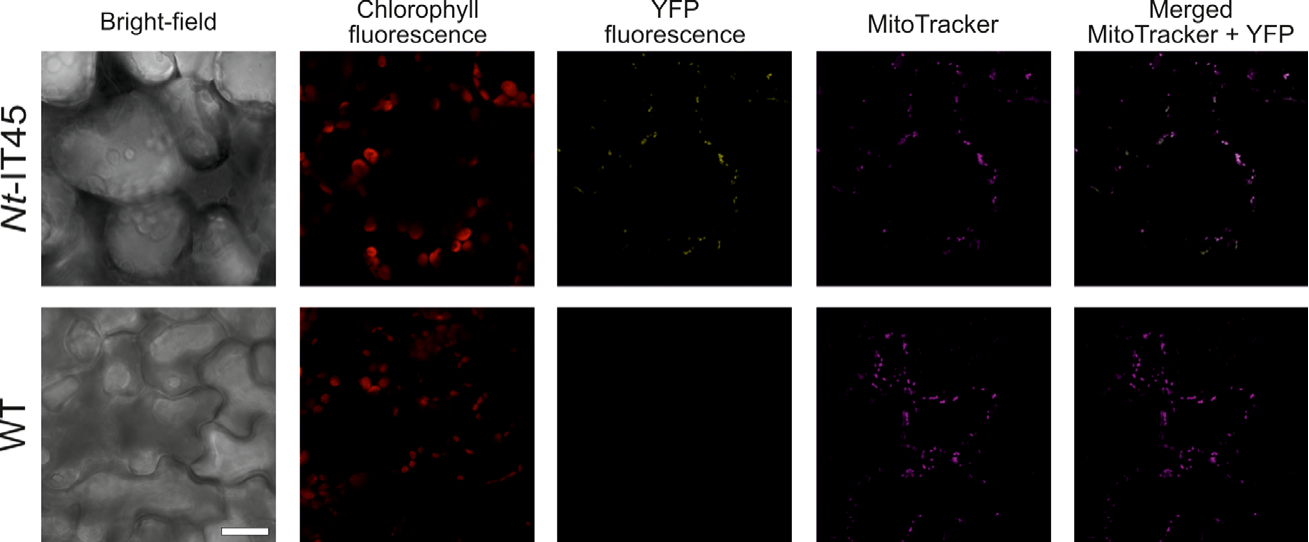
Confirmation of mitochondrial targeting directed by the CoxIV transit peptide from yeast. Transient transformation of *Nicotiana benthamiana* plants with vector pIT45 (Figure 2a) results in YFP fluorescence that localizes to mitochondria, as revealed by counterstaining with MitoTracker. The bright‐field image, the chlorophyll fluorescence, the YFP fluorescence, the MitoTracker fluorescence and the merged MitoTracker and YFP images are shown (from left to right). Sale bar: 20 μm.

To confirm that the yeast CoxIV transit peptide indeed targets the Sul protein to plant mitochondria, a vector carrying a transit peptide‐YFP fusion (pIT45; Figure 2a) was transiently transformed into tobacco cells. Confocal laser‐scanning microscopy revealed clear mitochondrial localization of the YFP fluorescence, as confirmed by counterstaining with MitoTracker (Figure 3).

### Specificity of sulfadiazine selection for mitochondria and efficacy of the optimized sulfadiazine selection system

Having obtained preliminary evidence for the specificity of the sulfadiazine selection system for mitochondria, a systematic approach was undertaken to (i) ultimately confirm the requirement for mitochondrial localization of the Sul resistance protein, (ii) determine the mode of action of the previously used Sul cassette containing the RbcS transit peptide, and (iii) assess the efficiency of the optimized sulfadiazine system by comparing it to the currently most efficient selectable marker gene for plant transformation, the kanamycin resistance gene *nptII*. To this end, a series of transformation vectors was constructed that is directly comparable in that the vectors differ only in the selectable marker gene and the targeting information it harbours (Figure 2a).

A relatively weak promoter, the promoter of the hydroxyperoxide lyase gene (*HPL*), was chosen to drive the expression of *sul* in all constructs and also the expression of *nptII* in control construct pIT36. The three *sul*‐containing vectors differ in the predicted subcellular localization of the Sul resistance protein: mitochondrial in pIT35, cytosolic in pIT41 and plastid in pIT42 (Figure 2a). As an additional control, a vector with a very strong *nptII* cassette under the control of the CaMV 35S promoter (pIT38) was also included. For the rapid visual detection of transgenic events obtained from antibiotic selection, all vectors also contain a YFP reporter (Figure 2a; Figure S3).

All constructs were transformed into tobacco cells using the biolistic method (Klein *et al*., 1988). Biolistic transformation was chosen, because it provides the opportunity to co‐transform two vectors, thus ensuring identical experimental conditions and facilitating direct comparison of the transformation efficiencies of two vectors. The co‐transformation approach was used to assess the efficiency of sulfadiazine versus kanamycin selection. To this end, vectors pIT35 and pIT38 were mixed in equimolar ratios and co‐bombarded. The transformed leaf pieces were split into two batches and selected on either 25 mg/L sulfadiazine or 50 mg/L kanamycin. In both selection conditions, primary resistant lines appeared after 3–4 weeks (Figures 2b and S4; Table 1).

**Table 1:** Statistics of biolistic transformation experiments to confirm the specificity of the sulfadiazine selection system for mitochondria and comparison of the efficacy of the optimized sulfadiazine selection with kanamycin selection

Vector	Selected explants	Selection (mg/L)	Primary resistant lines	Escapes	Number of resistant lines	Selection efficiency (%)^a^	Transformation efficiency^b^
pIT35	371	Sdz 25	172	12	160	93	0.43
pIT41	385	Sdz 25	14	14	0	0	0
pIT42	385	Sdz 25	20	15	5	25	0.012
pIT36	364	Kan 50	167	28	139	83	0.38
pIT38	252	Kan 50	131	20	111	85	0.44
pIT35 + pIT38^c^	154	Sdz 25	85	9	76	89	0.49
pIT35 + pIT38^c^	105	Kan 50	53	13	40	75	0.38

a Number of confirmed transformants divided by the number of primary resistant lines.

b Number of transformants divided by the number of selected explants.

c Co‐transformation and selection of half of the explants on kanamycin (Kan) and half on sulfadiazine (Sdz).

**Figure 4:**
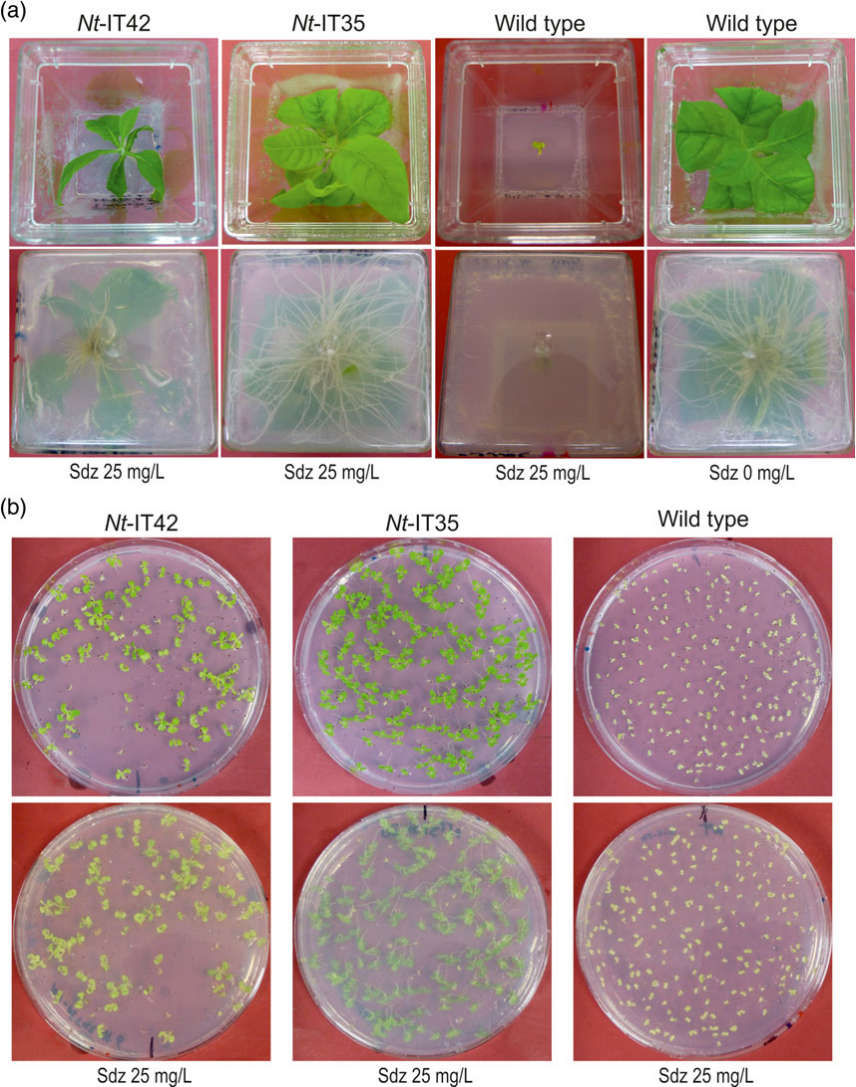
Comparison of growth and seed germination between *Nt*‐IT35 and *Nt*‐IT42 transgenic lines. (a) Growth behaviour of *Nt*‐IT35 (*sul* gene with a mitochondrial transit peptide sequence) and *Nt*‐IT42 (*sul* gene with a chloroplast transit peptide sequence) transgenic lines on medium with 25 mg/L sulfadiazine (Sdz). Photos were taken after 4 weeks. Note poor growth and delayed rooting of *Nt*‐IT42 plants. (b) Seed tests of *Nt*‐IT35 and *Nt*‐IT42 transgenic lines. Seed were germinated on medium with 25 mg/L sulfadiazine and photographed after 3 weeks. Note incomplete resistance of *Nt*‐IT42 seedlings as evidenced by their light green color.

Numerous resistant lines were obtained from transformation experiments with vectors pIT35, pIT36, pIT38 and the co‐transformation pIT35 + pIT38 (Figure 2a; Table 1). Regenerated shoots from the selection plates were rooted, propagated on medium containing 25 mg/L sulfadiazine or 50 mg/L kanamycin and then analyzed (Figure 4a; Figure S4; Table 1). A few lines were also obtained from transformation with vector pIT41 (*sul* without a transit peptide) and pIT42 (*sul* with the plastid *RbcS* transit peptide). However, none of the primary resistant shoots obtained with vector pIT41 showed durable resistance or developed roots in medium with sulfadiazine. A few primary resistant shoots (5 out of 20) obtained with vector pIT42 showed resistance and developed roots (Table 1). The inability to obtain transgenic lines with vector pIT41 and the low transformation frequency and poor sulfadiazine resistance levels obtained with vector pIT42 (Figure 4) lend strong support to the specificity of the sulfadiazine selection system for the mitochondrial compartment.

The results also suggest a likely explanation how sulfadiazine‐resistant transgenic lines were obtained in previous studies, in which the *sul* gene had been fused to a chloroplast transit peptide (*RbcS*) and the very strong CaMV35S promoter. We propose that massive overexpression of the Sul protein may have resulted in some mistargeting of Sul to mitochondria which was sufficient to confer phenotypic resistance to sulfadiazine. Low‐level dual targeting of overexpressed organellar proteins is common. A recent study tested 16 nucleus‐encoded organellar proteins by transient transformation assays and *in organello* protein import experiments. 10 of the 16 tested proteins were found to possess some dual targeting properties (Baudisch *et al*., 2014), suggesting that the selectivity of the targeting machineries for the two endosymbiotic organelles is limited and dual targeting (and low‐level mistargeting) are widespread.

In vector pIT42, we used the relatively weak *HPL* promoter to drive *sul* gene expression, thus potentially reducing the amount of protein that is mistargeted to mitochondria. Consistent with this idea, the number of transgenic lines obtained was substantially lower than in previous studies in which the *sul* gene was expressed from the CaMV35S promoter (Guerineau *et al*., 1990a; Wallis *et al*., 1996; Hadi *et al*., 2002; Thomson *et al*., 2011; Dal Bosco *et al*., 2004). Moreover, poor growth of *Nt*‐IT42 plants in the presence of sulfadiazine, delayed rooting, poor root growth (Figure 4a) and low‐level drug resistance of T1 seedlings (Figure 4b) provided further evidence against chloroplast‐targeted Sul conferring sulfadiazine resistance.

Comparison of the transformation experiments with vector pIT35 and pIT36 that harbour the sulfadiazine and kanamycin resistance genes in identical expression cassettes (Figure 2a), revealed that mitochondrially targeted Sul compares favourably to NptII as selectable marker for plant transformation (Table 1; Figure S4). Co‐transformation experiments with a mixture of vectors pIT35 and pIT38 demonstrated that the optimized (mitochondrially targeted) Sul is even a slightly more efficient selectable marker than the NptII overexpressed from the CaMV 35S promoter (Table 1).

### Efficient transformation of the alga *Chlamydomonas* with mitochondrially targeted Sul markers

Having established the *sul* gene as a highly efficient selectable marker for the transformation of the seed plant tobacco, we next wanted to test its potential suitability as a selectable marker for algal transformation. To this end, we determined the sensitivity of three laboratory strains of the model alga *Chlamydomonas reinhardtii* (CC‐503, CC‐1690, and the expression strain UVM11; Neupert *et al*., 2009) to sulfadiazine. 500 mg/L sulfadiazine was determined as minimum inhibitory concentration for the cell wall‐deficient strains UVM11 and CC‐503 (Figure S5), while the walled strain CC‐1690 required higher concentrations for complete growth inhibition (1200 mg/L; Figure S6).

**Figure 5:**
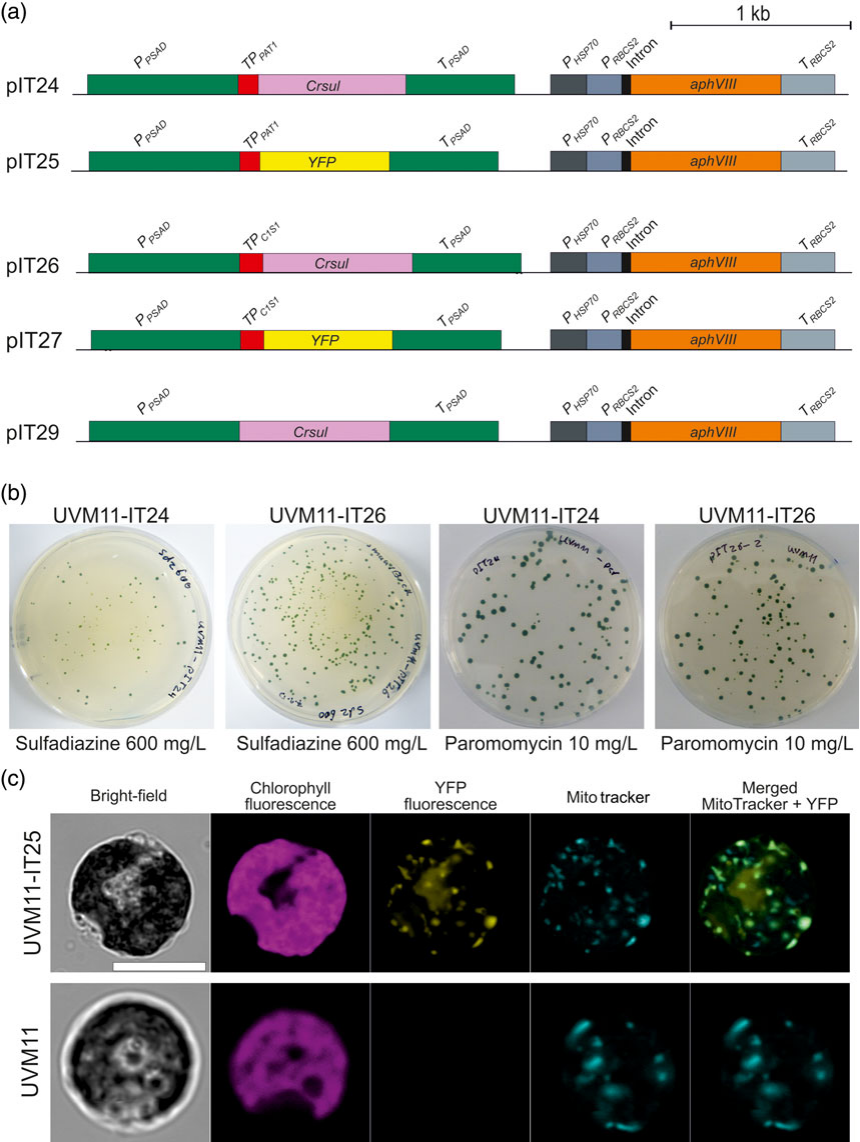
Optimization of the sulfadiazine selection system for transformation of *Chlamydomonas reinhardtii*. (a) Construction of nuclear transformation vectors. Vectors pIT24 and pIT25 contain the *sul* gene and *YFP* gene, respectively, fused to the phosphate acetyltransferase (PAT1) transit peptide to target the proteins to mitochondria. In pIT26 and pIT27, the genes are fused to the citrate synthase (C1S1) transit peptide that is also expected to target the proteins to mitochondria. Vector pIT29 contains the *sul* gene without a transit peptide. (b) Resistant colonies forming from transformation with vectors pIT24 and pIT26. (c) Confirmation of mitochondrial targeting. Transformation of algal expression strain UVM11 (Neupert *et al*., 2009; Barahimipour *et al*., 2015) with vector pIT25 results in strong YFP fluorescence that localizes to mitochondria. Mitochondrial localization was verified by staining with MitoTracker. The non‐transformed strain UVM11 is shown as negative control. Scale bars: 10 μm.

Next, a series of algal transformation vectors was constructed. In view of the importance of codon usage for efficient transgene expression in *Chlamydomonas* (Barahimipour *et al*., 2015), the *sul* coding region was codon‐optimized for the (highly GC‐rich) nuclear genome of *Chlamydomonas* (*Crsul* gene; Figure 5a). Two mitochondrial targeting signals were tested: the transit peptide of the phosphate acetyltransferase (PAT1) and the transit peptide of the citrate synthase (C1S1). To verify faithful protein import into the mitochondrial compartment, YFP fusions with both transit peptides were also constructed (Figure 5a). For comparison of transformation efficiencies in the same experiment, the paromomycin resistance gene *aphVIII* was incorporated in all vectors (Figure 5a). To confirm the requirement for mitochondrial location of Sul also in *Chlamydomonas*, an additional vector was constructed that encodes a Sul version without a transit peptide (pIT29; Figure 5a).

Nuclear transformation experiments in *Chlamydomonas* were performed using the glass bead‐assisted method and by electroporation (Kindle, 1990; Neupert *et al*., 2012). Sulfadiazine selection was attempted with vectors pIT24, pIT25 and pIT29, and paromomycin selection was used to obtain transgenic strains expressing the YFP fusions (pIT25 and pIT27; Figure 5a,b; Table 2). Paromomycin‐resistant colonies obtained with vectors pIT25 and pIT27 were analyzed by confocal laser‐scanning microscopy which showed clear mitochondrial localization of both YFP fusions, as confirmed by counterstaining with MitoTracker (Figure 5c). These data strongly suggest that both the PAT1 and the C1S1 transit peptides faithfully target proteins to algal mitochondria.

**Table 2:** Statistics of nuclear transformation experiments in *Chlamydomonas*. In the negative controls, no DNA was included in the transformation

Vector	Transformation method	Strain	Selection (mg/L)	Resistant colonies (average of 3 replicates)	Resistant after transfer to fresh medium	Confirmed by PCR
pIT24	Glass bead‐assisted	UVM11	Sdz 600	87	20/20	10/10
pIT24	Glass bead‐assisted	UVM11	Par 10	189	20/20	10/10
pIT26	Glass bead‐assisted	UVM11	Sdz 600	252	20/20	10/10
pIT26	Glass bead‐assisted	UVM11	Par 10	312	20/20	10/10
pIT29	Glass bead‐assisted	UVM11	Sdz 600	0	–	–
Negative control	Glass bead‐assisted	UVM11	Sdz 600	0	–	–
pIT24	Electroporation 800 V	UVM11	Sdz 600	80	20/20	10/10
pIT24	Electroporation 800 V	UVM11	Par 10	170	20/20	10/10
pIT26	Electroporation 800 V	UVM11	Sdz 600	280	20/20	10/10
pIT26	Electroporation 800 V	UVM11	Par 10	317	20/20	10/10
pIT29	Electroporation 800 V	UVM11	Sdz 600	0	–	–
Negative control	Electroporation 800 V	UVM11	Sdz 600	0	–	–
pIT24	Electroporation 1200 V	CC‐503	Sdz 600	187	20/20	10/10
pIT24	Electroporation 1200 V	CC‐503	Par 10	195	20/20	10/10
pIT26	Electroporation 1200 V	CC‐503	Sdz 600	270	20/20	10/10
pIT26	Electroporation 1200 V	CC‐503	Par 10	180	20/20	10/10
pIT29	Electroporation 1200 V	CC‐503	Sdz 600	0	–	–
Negative control	Electroporation 1200 V	CC‐503	Sdz 600	0	–	–

Interestingly, sulfadiazine selection also produced numerous transgenic algal clones in transformation experiments with vectors pIT24 and pIT26 (Figure 5b; Table 2). Transgenic clones were obtained with all three algal strains (Table 2; Figure S6). Resistance tests and molecular analyses confirmed the transgenic status of all sulfadiazine‐resistant clones (Table 2), suggesting that the selection is stringent enough to fully suppress the growth of non‐transgenic cells. Overall, the transformation efficiencies obtained with sulfadiazine selection were in the same range as those obtained with the established paromomycin selection (Table 2).

Consistent with the experiments in tobacco (Figure 2; Table 1), no resistant colonies were obtained with vector pIT29 that lacks a mitochondrial transit peptide sequence (Table 2). These results confirm that, also in *Chlamydomonas*, the folate pathway localizes to mitochondria and mitochondrial targeting of the Sul protein is required to confer sulfadiazine resistance. The mitochondrial *Crsul* cassettes in vectors pIT24 and pIT26 (Figure 5a) thus provide attractive alternative selectable markers for algal transformation.

## Discussion

The ability to produce transgenic cells is of equally great importance to both basic research and biotechnology. Although a number of selectable marker genes have been developed for the transformation of plants and algae (Hansen and Wright, 1999; Joersbo, 2001; Bock, 2015; Neupert *et al*., 2012; Doron *et al*., 2016), only relatively few marker genes are routinely used. This is because only few marker genes (i) allow the selection of transgenic events at high frequency, (ii) efficiently suppress the growth of untransformed cells, and (iii) are broadly applicable to a wider range of species. Why some markers are better than others is often not entirely clear. In the course of this work, we have determined the cause of the inefficiency of previously used sulfadiazine resistance markers and converted the *sul* gene into a highly efficient selectable marker gene for plant and algal transformation.

By both Agrobacterium‐mediated transformation and biolistic transformation, we have shown that, when targeted to the mitochondrial compartment, the Sul protein facilitates the selection of transgenic plants at frequencies that are similar or even higher than those achieved with the currently most efficient markers. Importantly, these high transformation frequencies are obtained with relatively low expression levels of the *sul* marker gene. By contrast, most currently used selectable markers rely on strong overexpression of the resistance protein, often driven by the CaMV 35S promoter (Figure 2; Table 1). The use of lowly expressed markers is potentially beneficial in that it lowers the metabolic burden imposed on the plant and reduces the probability of observing unintended phenotypic effects.

The need to develop new efficient selectable marker genes is particularly pressing in algae. Most algal species are currently not transformable and the lack of suitable selectable marker genes represents a major bottleneck in the development of transformation protocols for many groups of algae (Barahimipour *et al*., 2016). Due to the ubiquitous presence of the folate pathway in all plants and algae, the *sul* gene potentially provides a universal selectable marker gene for photosynthetic eukaryotes.

**Figure 6:**
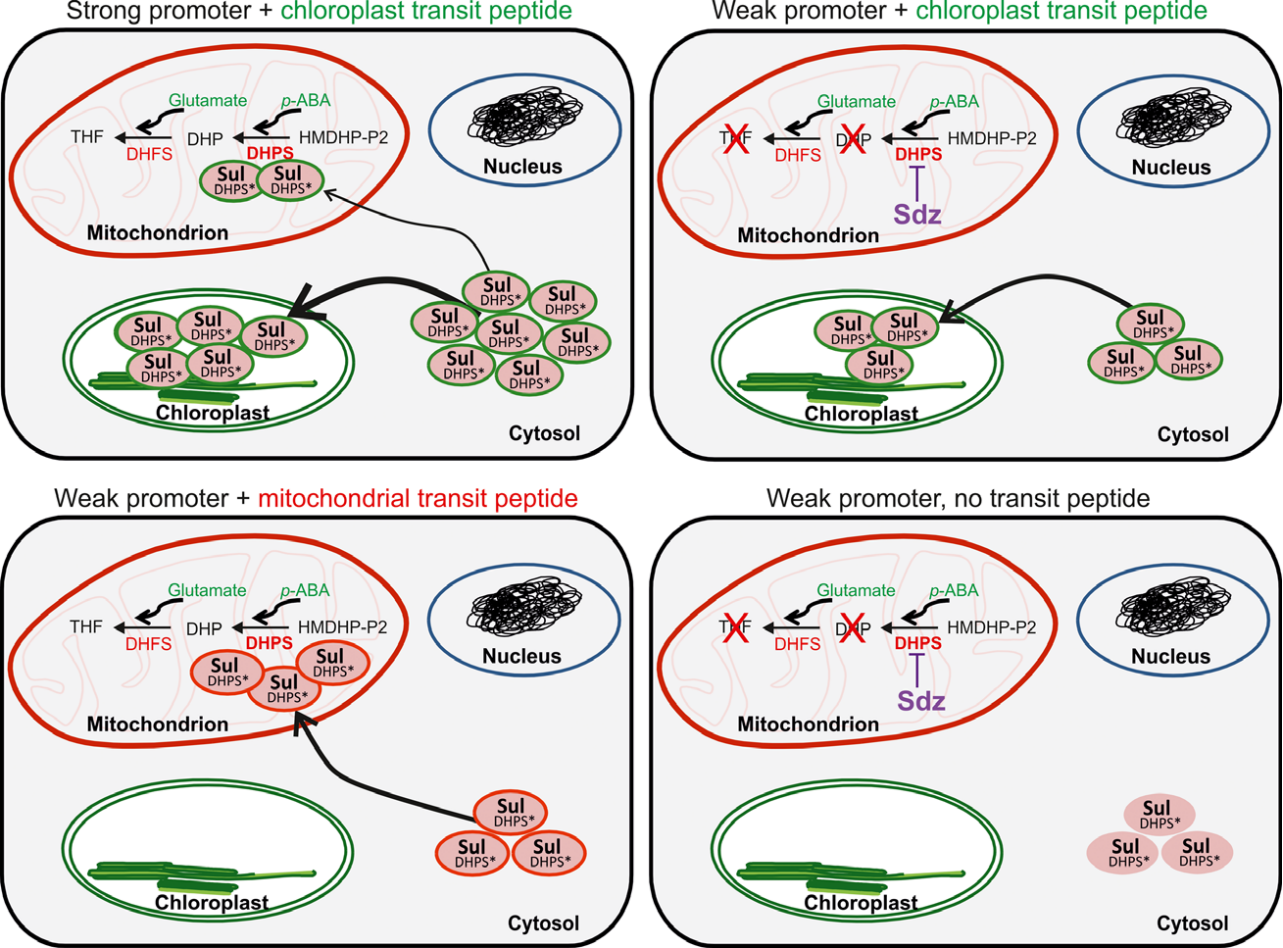
Model to explain the results of previous and current transgenic studies in the light of the specificity of the sulfadiazine selection system for mitochondria. See text for details. Sdz: sulfadiazine, *p*‐ABA:* p*‐amino benzoate, HMDHP‐P2: 6‐hydroxymethyldihydropterin pyrophosphate, DHP: dihydropteroate; DHPS: dihydropteroate synthase, DHFS: dihydrofolate synthase, THF: tetrahydrofolate.

Figure 6 summarizes the currently available data on the sulfadiazine selection system and its specificity for mitochondria. When Sul is massively overexpressed from previously published vectors targeting the protein to plastids (‘Strong promoter + chloroplast transit peptide’), some mistargeting of Sul to mitochondria may occur, allowing selection of transgenic plants, albeit with relatively low efficiency. When expression of plastid‐targeted Sul is low to moderate (‘Weak promoter + chloroplast transit peptide’), the protein is largely correctly targeted to chloroplasts where is does not confer protection from DHPS inhibition by sulfadiazine (Figure 6). In our optimized sulfadiazine selection system (‘Weak promoter + mitochondrial transit peptide’), the Sul protein is targeted to mitochondria where it efficiently catalyzes the DHPS reaction and, in this way, provides resistance to sulfadiazine. When not provided with any targeting information (‘Weak promoter, no transit peptide’), the Sul protein accumulates in the cytosol, where it cannot catalyze dihydropteroate synthesis (Table 1; Figure 6). Although, in the present study, the *sul* gene was exclusively used for nuclear transformation, due to the specific action of the Sul protein in the mitochondrial compartment, the gene may also provide a suitable selectable marker for the development of mitochondrial transformation. Mitochondrial transformation of plants has not been achieved yet, due to the lack of a suitable selectable marker gene that would specifically protect mitochondrial gene expression or metabolism from the inhibitory action of a mitochondria‐specific selection agent. Experiments are underway to test the suitability of *sul* cassettes as selectable marker genes for mitochondrial transformation in tobacco and *Chlamydomonas*.

In summary, our work reported here revealed the molecular cause of the inefficiency of sulfadiazine selection of transgenic plants with previous transformation vectors. Our optimized *sul*‐based vectors provide highly efficient selectable marker genes for the transformation of plants and algae that are likely applicable to a wide range of species. Finally, our work suggests sulfadiazine resistance genes as potential selectable markers for the development of mitochondrial transformation technology.

## Experimental procedures

### Plant material and growth conditions

Sterile tobacco (*Nicotiana tabacum* cv. Petit Havana) plants were grown in controlled environment chambers (light intensity: 50 μE/m/s; photoperiod: 16 h light, 8 h darkness; day temperature: 25°C, night temperature: 22°C) on agar‐solidified MS medium (Murashige and Skoog, 1962) containing 30 g/L sucrose. For biolistic transformation experiments, young leaves were harvested from 4‐week‐old plants raised from seeds. Regenerated transgenic shoots were rooted and propagated in MS medium supplemented with 25 mg/L sulfadiazine (Sigma) or 50 mg/L kanamycin (Duchefa). For seed production and inheritance assays, rooted transgenic plants were transferred to soil and grown to maturity under standard greenhouse conditions. Seeds were harvested and assayed for Mendelian inheritance by germination on sulfadiazine‐containing MS medium (20 or 25 mg/L sulfadiazine).

### Cultivation of *Agrobacterium tumefaciens*

*A. tumefaciens* strain GV2260 was used for nuclear transformation of tobacco plants. Bacteria were grown in liquid cultures for 40 h at 28°C on a rotary shaker at 180 rpm, in YEB medium containing rifampicin (100 mg/L), carbenicillin (50 mg/L) and kanamycin (100 mg/L). For growth of bacteria in Petri dishes, YEB medium was solidified with 15 g/L agar and supplemented with the appropriate antibiotics for selection.

### Cultivation of *Chlamydomonas reinhardtii*

*C. reinhardtii* expression strain UVM11 (Neupert *et al*., 2009) and the wild‐type‐like strains CC‐1690 and CC‐503 were used for transformation experiments. Expression strain UVM11 was obtained by UV light‐induced mutagenesis and selection for improved transgene expression (Neupert *et al*., 2009). Algal cells were cultivated mixotrophically either in liquid Tris‐acetate‐phosphate (TAP) medium or on agar‐solidified TAP medium at 22°C under continuous light (light intensity 50–100 μE/m/s; Harris, 1989; Neupert *et al*., 2012), unless otherwise stated.

### Construction of transformation vectors

pIT15 is a nuclear transformation vector that contains the *sul* gene fused to the 5 N‐terminal codons of the mitochondrial *rps10* gene and the mitochondrial transit peptide sequence derived from the *CoxIV* gene of the yeast *Saccharomyces cerevisiae*. Transformation vectors were assembled using the pORE‐E2 plasmid (Coutu *et al*., 2007) as backbone. The *sul* gene was amplified from the p35S*sul* plasmid (derived from plasmid pSEX001‐VS; Reiss *et al*., 1996) by PCR with primers FW‐cox4‐4rps10‐sul (also containing the first five codons of *rps10*) and RE‐KpnI‐sul (Table S2). The transit peptide of the yeast cytochrome oxidase subunit IV (*CoxIV* gene; Hurt *et al*., 1985) was amplified by PCR from a transgenic *Arabidopsis thaliana* line (mCherry line; Nelson *et al*., 2007) with primers FW‐BamHI‐cox4 and RE‐4rps10‐cox4 (Table S2). The two PCR fragments were fused by amplification with primers FW‐BamHI‐cox4 and RE‐KpnI‐sul and the resulting product was cloned as BamHI/KpnI restriction fragment into vector pORE‐E2. The *sul* gene in this plasmid is driven by the *A. thaliana* hydroperoxide lyase promoter (P_*HPL*_) and the nopaline synthase terminator (T_*NOS*_).

Vector pIT17 is similar to pIT15, but lacks the five N‐terminal codons of *rps10*. Vector pIT18 contains the *sul* gene without a transit peptide. It was generated by amplification of the *sul* gene with primers FW‐BamHI‐sul and RE‐KpnI‐sul (Table S2) and cloning of the PCR product as a BamHI/KpnI restriction fragments into vector pORE‐E2. The same promoter and terminator were used for the *sul* cassette as in pIT15. Vector pIT35 contains the *sul* gene (driven by P_*HPL*_ and T_*NOS*_) fused to the *CoxIV* mitochondrial transit peptide cloned into vector pORE‐O4 (Coutu *et al*., 2007). The vector additionally contains a *YFP* cassette as reporter. For construction of the *YFP* cassette, the CaMV 35S promoter was amplified by PCR with primers oIT64_XhoI_P35s and oIT75_3_35S (Table S2). The *YFP* coding region and the CaMV 35S terminator were obtained from a *YFP*‐containing pGreen vector (Stegemann and Bock, 2009). The two fragments were fused by PCR with primers oIT64_XhoI_P35s and oIT65_NcoI_TCaMV (Table S2). The *YFP* cassette was then cloned as a NcoI/XhoI restriction fragment into a derivative of pIT17. Vector pIT36 is similar to pIT35, but contains *nptII* instead of *sul*. Vector pIT38 contains the *nptII* gene driven by the ENTCUP2 promoter and the *YFP* cassette. The ENTCUP2 promoter was amplified by PCR with primers oIT84_XhoI_ 5Cup and oIT85_XbaI_3Cup from vector pORE‐E3 (Coutu *et al*., 2007) and then cloned as an NcoI/XhoI restriction fragment into the a derivative of pIT36. Vector pIT41 contains the *sul* gene without an organellar transit peptide, generated by amplification of the *sul* gene from vector pIT35 with primers oIT80_Bam_sulF and oIT68_Asc_sulR (Table S2) and insertion as AscI/BamHI restriction fragment into pIT40. Vector pIT42 was constructed as a control to analyze mistargeting of Sul to the plastid. In this vector, *sul* is fused to the pea (*Pisum sativum*) Rubisco small subunit (*RbcS*) transit peptide for protein targeting to the chloroplast. The *sul* gene with the *RbcS* transit peptide sequence was amplified from an existing cassette (Reiss *et al*., 1996) with primers oIT81_Bam_Cpsul and oIT68_Asc_sulR and then cloned as AscI/BamHI restriction fragment into pIT40.

pIT24 is a nuclear transformation vector for *C. reinhardtii*. The vector contains the *sul* gene codon‐optimized for *Chlamydomonas* and fused to the phosphate acetyltransferase (PAT1) transit peptide, known to target proteins to algal mitochondria (Yang *et al*., 2014). The *sul* gene in pIT24 is flanked by the promoter and terminator of the *PSAD* gene from *C. reinhardtii* (Fischer and Rochaix, 2001). The vector was constructed based on pRMB27 (Barahimipour *et al*., 2016), which contains the *aphVIII* gene (conferring resistance to paromomycin) under the control of the hybrid *HSP70A‐RBCS2* promoter and the *RBCS2* terminator (Schroda *et al*., 2000). The *sul* gene was inserted as Ndel/EcoRI fragment into the similarly cut pRMB27. Vector pIT25 is identical to pIT24, except that the *sul* gene was replaced with *YFP*. A codon‐optimized *YFP* was amplified by PCR with primers oIT45_Mlu1‐5YFP and oIT46_EcoRI‐3YFP (Table S2) from plasmid pRMB12 (Barahimipour *et al*., 2015). The amplification product was digested with the restriction enzymes MluI and EcoRI, and then cloned into vector pIT24 cut with the same enzymes. pIT26 is derived from pIT24 and contains the codon‐optimized *sul* gene fused to the citrate synthase (CIS1) transit peptide, known to target proteins to *C. reinhardtii* mitochondria (Matsuo *et al*., 2011). The CIS1 transit peptide was amplified with primers oIT43_NdeI‐3CIS1 and oIT44_Mlu1‐5CIS1 (Table S2), then cut with MluI and NdeI and cloned into vector pIT24. Vector pIT27 was derived from pIT26 by replacing the *sul* gene with the codon‐optimized *YFP* from pRMB12 (Barahimipour *et al*., 2015) as MluI/EcoRI restriction fragment. Vector pIT29 contains the *sul* gene without a transit peptide, amplified by PCR with primers oIT41_NdeI‐3Crsul and oIT42_EcoRI‐5Crsul (Table S2), digested with NdeI and EcoRI, and then cloned into the similarly cut pIT24.

Vector pIT45 was constructed to visualize mitochondrial localization of YPF in plant cells using the yeast mitochondrial transient peptide. The vector contains the *YFP* gene fused to the mitochondrial transit peptide sequence from the *CoxIV* gene of the yeast *Saccharomyces cerevisiae* and was assembled using the pORE‐E2 plasmid (Coutu *et al*., 2007) as backbone. The *YFP* gene was amplified from plasmid pIT35 by PCR with primers OIT122_Fw_EYFP and OIT123_Re_EYFP (Table S2). The transit peptide of the yeast cytochrome oxidase subunit IV (*CoxIV* gene; Hurt *et al*., 1985) was amplified by PCR from a transgenic *Arabidopsis thaliana* line (mCherry line; Nelson *et al*., 2007) with primers OIT120_Fw_cox4 and OIT121_Re_cox4 (Table S2). The two PCR fragments were fused by amplification with primers OIT120_Fw_cox4 and OIT123_Re_EYFP and the resulting product was cloned as BamHI/KpnI restriction fragment into vector pORE‐E2.

### Transformation of *Nicotiana*

Nuclear transformations by biolistic bombardment of *N. tabacum* leaves was performed with 0.6 μm gold particles (BioRad, Munich, Germany) according to published protocols (Klein *et al*., 1988; Ruf and Bock, 2011). Bombardment experiments were conducted with a DuPont PDS‐1000/He biolistic gun (BioRad) with the hepta adaptor setup. For co‐transformation experiments, vectors pIT35 (carrying *sul*) and pIT38 (carrying *nptII*) were mixed in equimolar ratios. The bombarded leaves were cut into small pieces (~5 × 5 mm) which were placed onto the surface of an MS‐based selective regeneration medium containing the appropriate antibiotic (sulfadiazine at 20 or 25 mg/L, kanamycin at 50 mg/L). Selected antibiotic‐resistant lines were rooted, propagated on the same medium (containing 25 mg/L sulfadiazine or 50 mg/L kanamycin), and then transferred to soil and grown to maturity under standard greenhouse conditions.

Agrobacterium‐mediated transformation of *Nicotiana tabacum* leaf discs was performed with *Agrobacterium tumefaciens* strain pGV2260 following standard protocols (Bevan, 1984).

For transient expression, *Agrobacterium* strain GV2260 was grown at 28°C at 180 rpm for 24 hr in LB medium with kanamycin (50 mg/L) and rifampicillin (50 mg/L). Cells were harvested by centrifugation for 30 min at 4000 *g* at room temperature and then resuspended in 10 mm MES buffer containing 10 mm MgCl_2_ and 100 mm acetosyringone to a final OD_600_ of 0.4 followed by incubation at room temperature and 70 rpm for 120 min. *Agrobacterium* strains harboring the transformation plasmid were infiltrated into leaves of 4‐week‐old *Nicotiana benthamiana* plants using a 1 mL syringe. The infiltrated plants were then grown under greenhouse conditions in low light for 2 days, followed by harvesting of leaf pieces for microscopic analysis.

### Transformation of *Chlamydomonas reinhardtii*

Nuclear transformation of *C. reinhardtii* was performed by glass bead‐assisted DNA delivery (Kindle, 1990; Neupert *et al*., 2012) or by electroporation. Typically, samples of 1 μg linearized plasmid were used for transformation of a suspension of 3.3 × 10^8^ cells/mL. Electroporation of strain UVM11 was performed at 800 V for 5 ms using a Multiporator (Eppendorf). For strains CC‐503 and CC‐1690, 1200 V was used. Transformed cells were spread on selection plates containing agar‐solidified TAP medium supplemented with the appropriate antibiotics.

### Isolation of genomic DNA from plants and algae, and PCR analyses

Total plant DNA was isolated from fresh leaf material using a cetyltrimethylammonium bromide (CTAB)‐based protocol (Doyle and Doyle, 1990). Total genomic DNA from *Chlamydomonas* was extracted according to published protocols (Schroda *et al*., 2000). Samples of 100 ng genomic DNA were used as template for PCR assays to identify transformants that have the complete *sul* cassette integrated into their nuclear genome. PCR assays were conducted with two primer pairs (Neupert *et al*., 2009) and algal strains yielding both PCR products were selected as positive clones. Primer pair oIT101_FpSAD_NCr (5′‐CTCGGGGGGAGGTTTCCT‐3′) and oIT100_Rsul_NcCr (5′‐ATGTCGGGGTACAGGGCG‐3′) amplifies the 3′ end of the *PSAD* promoter upstream of the coding region of the *sul* gene (580 bp amplicon), and primers oIT98_Fsul_NcCr (5′‐ATGGTGACCGTGTTCGGCA‐3′) and oIT99_Rsul_NcCr (5′‐TTAGGCGTGGTCCAGGCCG‐3′) amplify the *sul* coding region (840 bp amplicon).

### Microscopic analyses

YFP fluorescence in algal cells was detected with a confocal laser‐scanning microscope (TCS SP5; Leica, Wetzlar, Germany) using an argon laser for excitation (514 nm), a 524–561 nm filter for detection of YFP fluorescence, and a 665–706 nm filter for detection of chlorophyll fluorescence. For MitoTracker staining, 2 mL of an algal culture (approximately 3 × 10^6^ cells) was gently centrifuged (1100 *g* for 1 min), the supernatant removed, the dye added (1 mm MitoTracker^TM^ Orange CMTMRos; Invitrogen), and the sample was gently resuspended and incubated for 20 min at room temperature to allow uptake of the dye into the cells. 10 μL of the sample was placed on a glass slide and imaged using a 577–642 nm filter for detection of MitoTracker fluorescence with a confocal laser‐scanning microscope (TCS SP5, Leica). For staining of leaf cells, 2 μL of the dye were added to 2 mL of water containing a leaf piece of the transformed plant (0.3 × 0.3 mm), and the sample was incubated for 30–45 min in the dark at room temperature under gentle shaking to allow uptake of the dye into the cells. Leaf samples were then placed on a glass slide and imaged using a 566–596 nm filter for detection of MitoTracker fluorescence with a confocal laser‐scanning microscope (TCS SP8; Leica). YFP fluorescence in plant cells was detected with a confocal laser‐scanning microscope (TCS SP8) using an argon laser for excitation (514 nm), a 520–555 nm filter for detection of YFP fluorescence, and a 655–700 nm filter for detection of chlorophyll fluorescence.

## Conflict of interest

The authors declare no financial or commercial conflict of interest.

## Supporting information


**Figure S1** Sulfadiazine sensitivity tests in tobacco to determine the effective selection window.
**Figure S2** Wild‐type‐like phenotype of transgenic plants generated with the mitochondrially targeted sulfadiazine resistance protein and seed assays to confirm Mendelian inheritance.
**Figure S3** Detection of YFP fluorescence in transgenic tobacco cells.
**Figure S4** Comparison of the efficacy of the optimized sulfadiazine selection with the kanamycin selection system.
**Figure S5** Sulfadiazine sensitivity tests with strains of the unicellular green alga *Chlamydomonas reinhardtii* to determine the effective selection window.
**Figure S6** Transformation of the walled wild‐type strain CC‐1690 of *Chlamydomonas reinhardtii* with the *sul* vector pIT26.
**Table S1** Statistics of biolistic transformation experiments to confirm the specificity of the sulfadiazine selection system for mitochondria and to optimize the sulfadiazine selection for Agrobacterium‐mediated transformation.
**Table S2** PCR primers used for construction of transformation vectors.
