# Supplementary material for: A highly efficient sulfadiazine selection system for the generation of transgenic plants and algae
Source: Plant Biotechnol J. 2018 Sep 13;17(3):638–49. doi: 10.1111/pbi.13004 (PMC6381783; doi:10.1111/pbi.13004)
Supplement: Supplementary file 1 — Figure S1 Sulfadiazine sensitivity tests in tobacco to determine the effective selection window. Figure S2 Wild‐type‐like phenotype of transgenic plants generated with the mitochondrially targeted sulfadiazine resistance protein and seed assays to confirm Mendelian inheritance. Figure S3 Detection of YFP fluorescence in transgenic tobacco cells. Figure S4 Comparison of the efficacy of the optimized sulfadiazine selection with the kanamycin selection system. Figure S5 Sulfadiazine sensitivity tests with strains of the unicellular green alga Chlamydomonas reinhardtii to determine the effective selection window. Figure S6 Transformation of the walled wild‐type strain CC‐1690 of Chlamydomonas reinhardtii with the sul vector pIT26. Table S1 Statistics of biolistic transformation experiments to confirm the specificity of the sulfadiazine selection system for mitochondria and to optimize the sulfadiazine selection for Agrobacterium‐mediated transformation. Table S2 PCR primers used for construction of transformation vectors. [file PBI-17-638-s001.pdf]

SUPPLEMENTARY MATERIALS

A highly efficient sulfadiazine selection system for the generation of transgenic plants and algae

Iman Tabatabaei, Cristina Dal Bosco, Marta Bednarska, Stephanie Ruf, Jörg Meurer and Ralph Bock

Table S1 Statistics of the nuclear transformation experiments to confirm the specificity of the sulfadiazine (Sdz) selection system for mitochondria, and to optimize the sulfadiazine selection for Agrobacterium-mediated transformation.

Vector	Selection (mg/L Sdz)	Selected explants	Primary resistant shoots	Escapes	Transgenic lines	Selection efficiency (%)*
pIT15	20	285	126	23	103	82
pIT17	20	276	120	22	98	82
pIT18	20	295	42	42	0	0

* Number of confirmed transformants divided by the number of primary resistant lines

Table S2 PCR primers used for construction of transformation vectors. Restriction endonuclease recognition sites are underlined.

Oligonucleotide	Sequence (5' → 3')
FW-cox4-4rps10-sul	ACCACCAAGATAGTGACGGTGTTCGGC
RE-KpnI-sul	GTGGTACCCTAGGCATGATCTAACCCTCG
FW-BamHI-cox4	GTGGGATCCATGCTTTCACTACGTCAATCTATAAG
RE-4rps10-cox4	CCTATCTTGGTGGTGGGTTTTTGCTGAAGCA
oIT64_XhoI_P35s	ATATCTCGAGGTACCCCTACTCCAAAAATG
oIT75_3_35S	GACCGGTGGATCCTCTAGAAAGCTT
oIT65_NcoI_TCaMV	ATACCATGGGATCTGGATTTTAGTACTGGAT
oIT80_Bam_sulF	AGGATCCATGGTGACGGTGTTCG
oIT68_Asc_sulR	AGGCGCGCCCTAGGCATGATCTAACCCTCG
oIT81_Bam_Cpsul	AGGATCCATGGGCCCATTCGG
oIT45_Mlu1-5YFP	AAAACGCGTATGGTGAGCAAGGG
oIT46_EcoRI-3YFP	GGCAGAATTCTTACTTGATCAGCTCGTC
OIT120_Fw_cox4	GTGGGATCCATGCTTTCACTACGTCAATCTATAAG
OIT121_Re_cox4	GCCCTTGCTCACCATGGGTTTTTGCTGAAGCAGATATCT
OIT122_Fw_EYFP	CTTCAGCAAAAACCCATGGTGAGCAAGGGCGAGG
OIT123_Re_EYFP	ACGGTACCTTACTTGTACAGCTCGTCCA

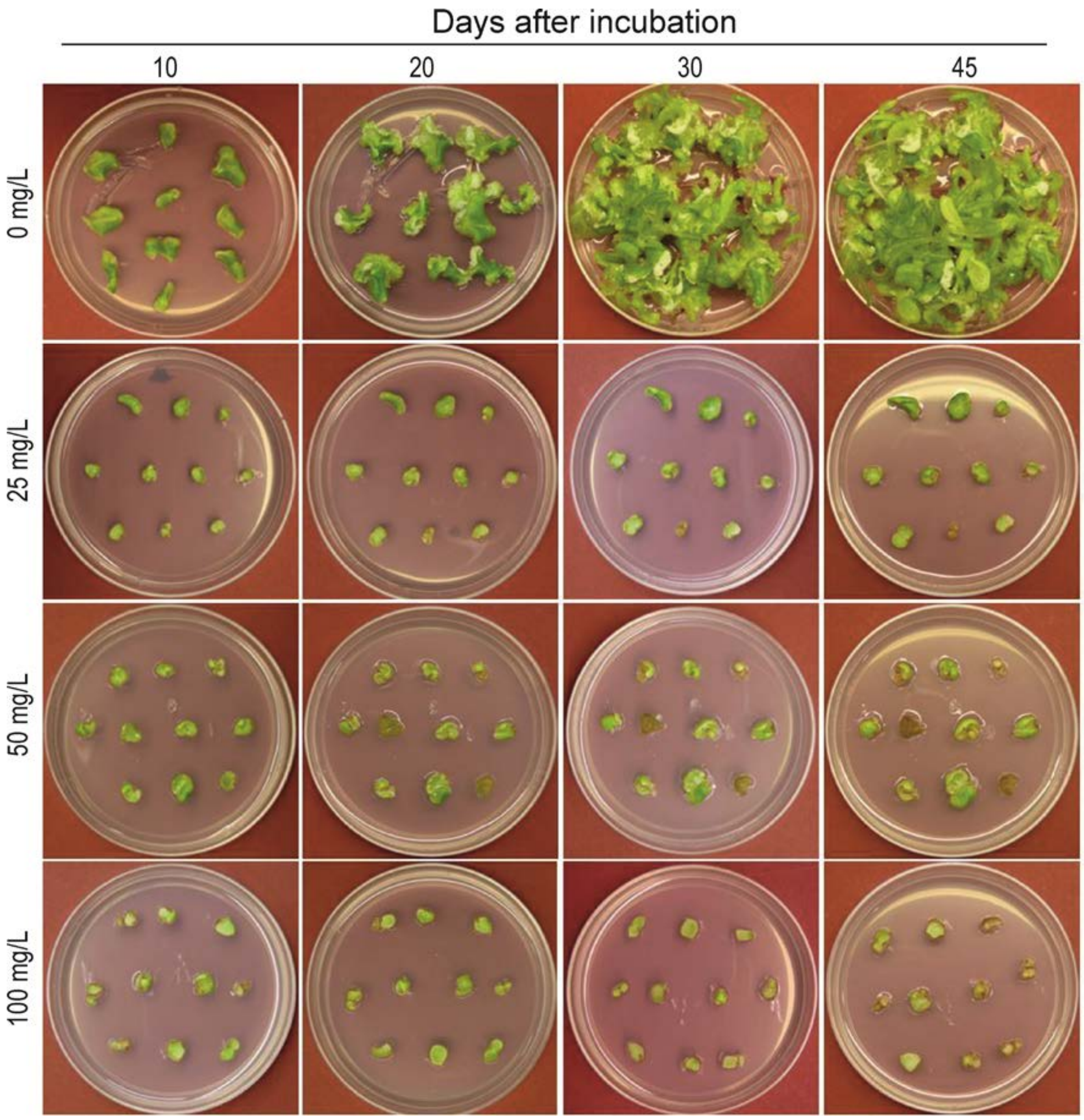

Figure S1 Sulfadiazine sensitivity tests in tobacco to determine the effective selection window.

The growth behavior of leaf pieces exposed to regeneration media containing different concentrations of sulfadiazine was analyzed after 10, 20, 30 and 45 days of incubation.

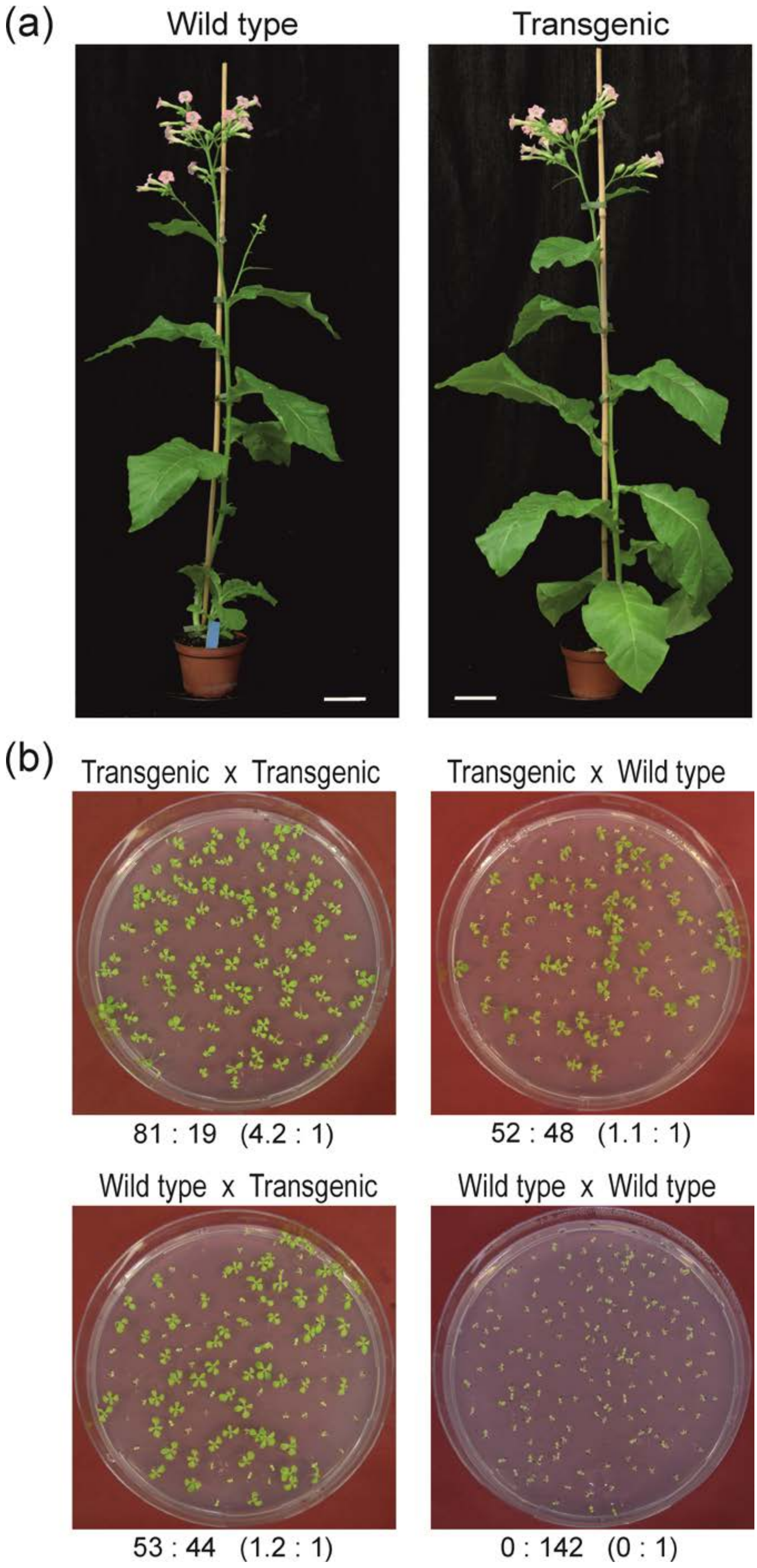
(a)
Wild type
Transgenic
(b)
Transgenic x Transgenic
Transgenic x Wild type
81 : 19 (4.2 : 1)
52 : 48 (1.1 : 1)
Wild type x Transgenic
Wild type x Wild type
53 : 44 (1.2 : 1)
0 : 142 (0 : 1)

Figure S2 Wild-type-like phenotype of transgenic plants generated with the mitochondrially targeted sulfadiazine resistance protein and seed assays to confirm Mendelian inheritance.

(**a**) Transgenic plants with the sulfadiazine resistance gene grew like wild-type plants in the greenhouse and produced normal amounts of seeds. An *Nt*-pIT17 plant is shown as an example. Scale bars: 10 cm.

(**b**) Reciprocal crosses to confirm stable inheritance of the sulfadiazine resistance. Crosses of transgenic plants with wild-type plants result in the expected 1:1 segregation in the next generation.

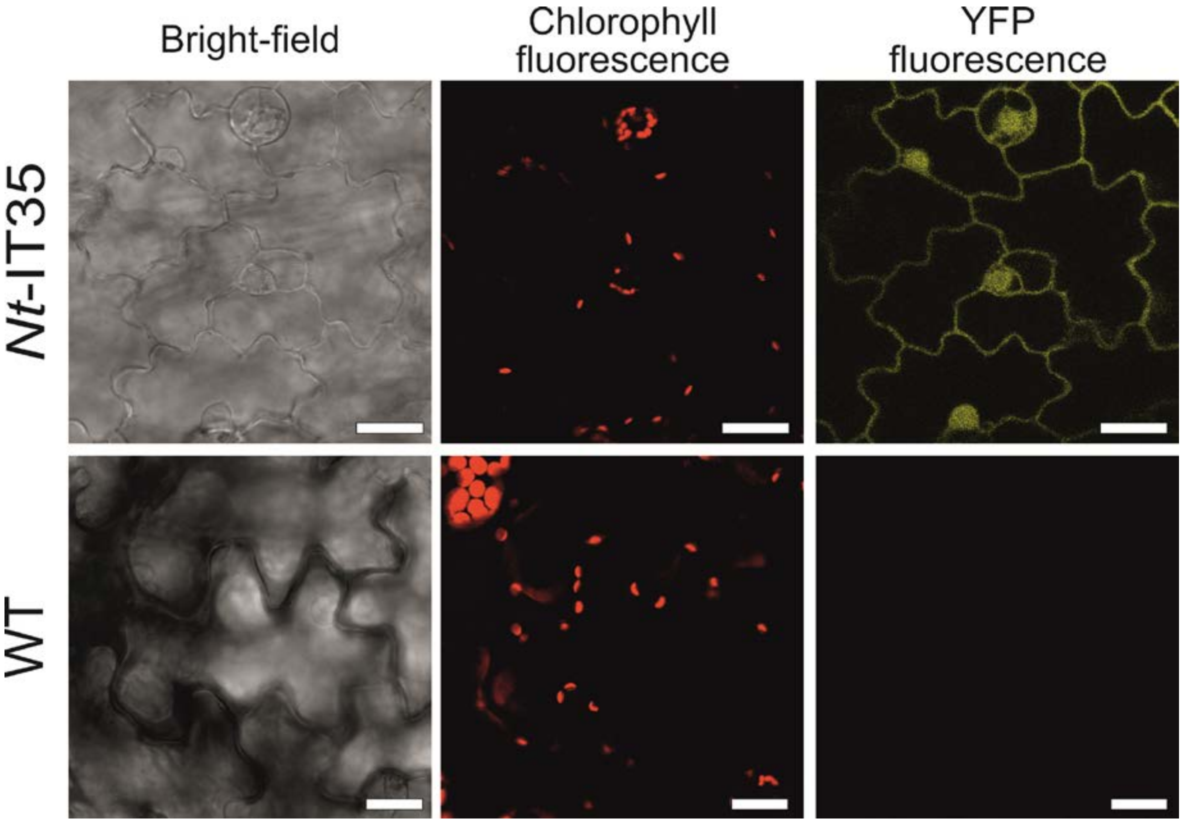

Figure S3 Detection of YFP fluorescence in transgenic tobacco cells. Fluorescence in leaf epidermal cells of plants stably transformed with construct pIT35 (Figure 2a; Figure 3a) is exemplarily shown. As expected, the YFP accumulates in the nucleus and the cytosol. The untransformed wild type (WT) is shown as a control. The bright-field image, the chlorophyll fluorescence and the YFP fluorescence are displayed from left to right. Sale bars: 20 μm.

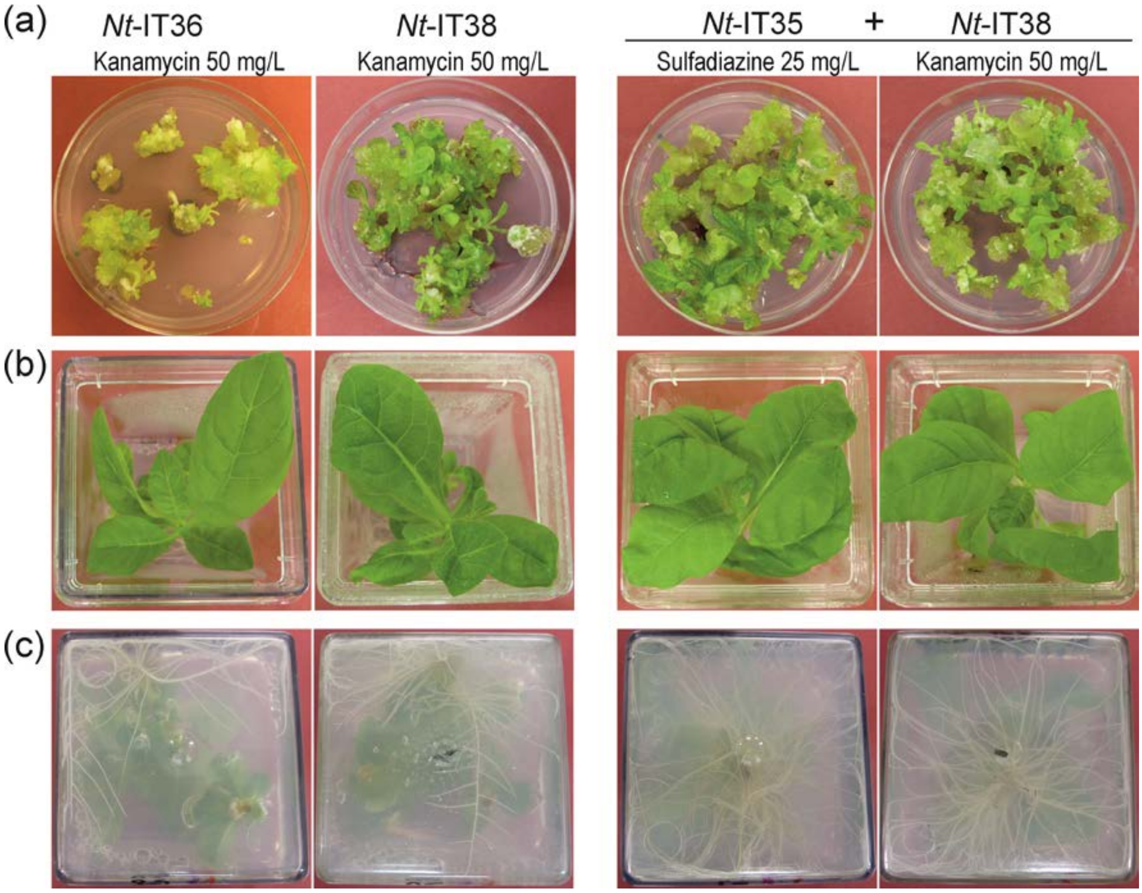

Figure S4 Comparison of the efficacy of the optimized sulfadiazine selection with the kanamycin selection system.

(**a**) Regeneration of primary sulfadiazine (*Nt*-pIT35) and kanamycin (*Nt*-IT36 and *Nt*-IT38) resistant cells. The photos were taken 5 weeks after transformation. *Nt*-pIT35 + *Nt*-IT38 denotes the co-transformation experiment (cf. Table 1).

(**b**) Growth of sulfadiazine and kanamycin-resistant shoots after transfer to rooting medium with 25 mg/L sulfadiazine or 50 mg/L kanamycin. Photos were taken after 5 weeks.

(**b**) Rooting of transgenic plants in the presence of the selection agents.

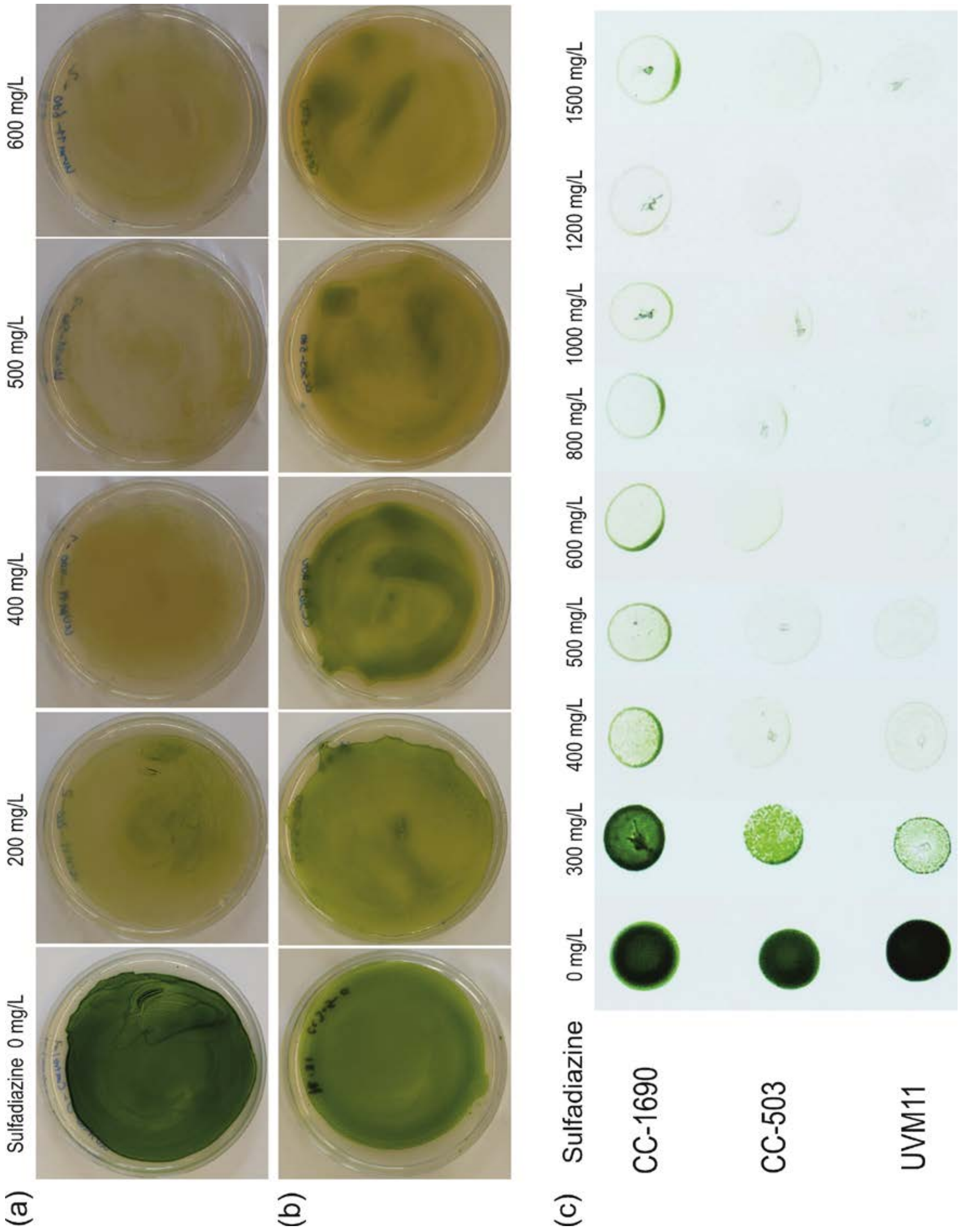

Figure S5 Sulfadiazine sensitivity tests with strains of the unicellular green alga *Chlamydomonas reinhardtii* to determine the effective selection window.

(**a**) Sulfadiazine sensitivity of expression strain UVM11.

(**b**) Sulfadiazine sensitivity of strain CC-503. Algae were plated on TAP medium containing different concentrations of sulfadiazine and photographed after 2 weeks of incubation.

(**c**) Sulfadiazine sensitivity of strains CC-1690, CC-503 and UVM11, as determined by drop tests (of algal cultures with a cell density of 10^7 cells mL^{-1}) medium with different concentrations of sulfadiazine. CC-1690 has a slightly higher tolerance to the drug, presumably because it forms a robust cell wall. (Note that CC-503 and UVM11 are cell wall-deficient strains.)

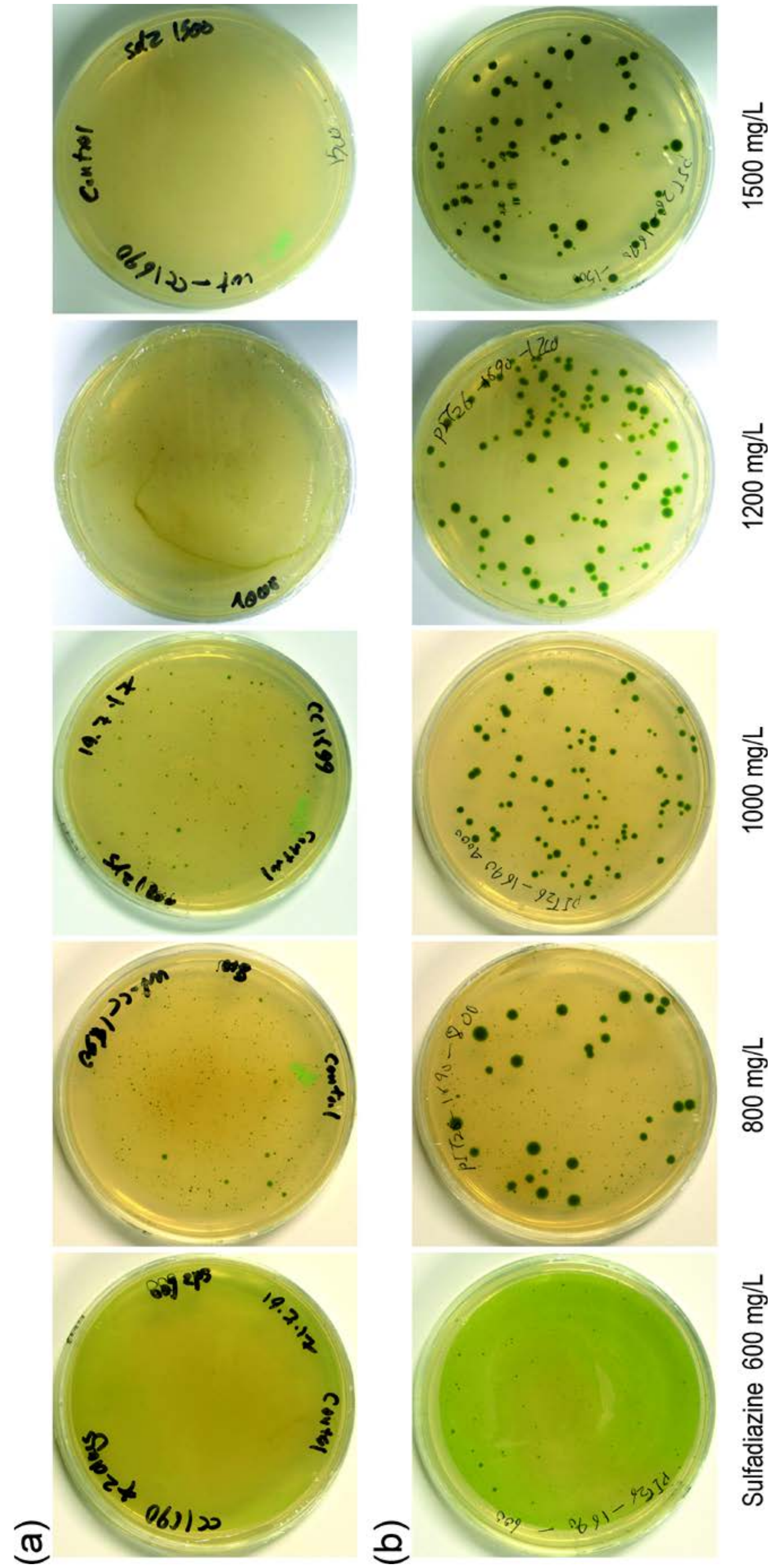
(a)
(b)
Sulfadiazine 600 mg/L
800 mg/L
1000 mg/L
1200 mg/L
1500 mg/L

Figure S6 Transformation of the walled wild-type strain CC-1690 of *Chlamydomonas reinhardtii* with the *sul* vector pIT26. Transformed algal clones were selected on different sulfadiazine concentrations.

(**a**) Negative control (transformation experiment without DNA).

(**b**) Transformation with pIT26.

List of legends

Figure 1 Construction of nuclear transformation vectors for optimization of the sulfadiazine selection system and generation of transgenic tobacco lines.

Figure 2 Construction of nuclear transformation vectors to confirm the specificity of the sulfadiazine selection system for mitochondria and compare the efficiency of the optimized sulfadiazine selection system with that of the kanamycin selection system.

Figure 3 Confirmation of mitochondrial targeting directed by the CoxIV transit peptide from yeast.

Figure 4 Comparison of growth and seed germination between *Nt*-IT35 and *Nt*-IT42 transgenic lines.

Figure 5 Optimization of the sulfadiazine selection system for transformation of *Chlamydomonas reinhardtii*.

Figure 6 Model to explain the results of previous and current transgenic studies in the light of the specificity of the sulfadiazine selection system for mitochondria.

Table 1 Statistics of biolistic transformation experiments to confirm the specificity of the sulfadiazine selection system for mitochondria and comparison of the efficacy of the optimized sulfadiazine selection with kanamycin selection.

Table 2 Statistics of nuclear transformation experiments in *Chlamydomonas*. In the negative controls, no DNA was included in the transformation.

Table S1 Statistics of the nuclear transformation experiments to confirm the specificity of the sulfadiazine (Sdz) selection system for mitochondria, and to optimize the sulfadiazine selection for Agrobacterium-mediated transformation.

Table S2 PCR primers used for construction of transformation vectors. Restriction endonuclease recognition sites are underlined.

Figure S1 Sulfadiazine sensitivity tests in tobacco to determine the effective selection window.

Figure S2 Wild-type-like phenotype of transgenic plants generated with the mitochondrially targeted sulfadiazine resistance protein and seed assays to confirm Mendelian inheritance.

Figure S3 Detection of YFP fluorescence in transgenic tobacco cells.

Figure S4 Comparison of the efficacy of the optimized sulfadiazine selection with the kanamycin selection system.

Figure S5 Sulfadiazine sensitivity tests with strains of the unicellular green alga *Chlamydomonas reinhardtii* to determine the effective selection window.

Figure S6 Transformation of the walled wild-type strain CC-1690 of *Chlamydomonas reinhardtii* with the *sul* vector pIT26.
